# Reductive Evolution of the Mitochondrial Processing Peptidases of the Unicellular Parasites *Trichomonas vaginalis* and *Giardia intestinalis*

**DOI:** 10.1371/journal.ppat.1000243

**Published:** 2008-12-19

**Authors:** Ondřej Šmíd, Anna Matušková, Simon R. Harris, Tomáš Kučera, Marián Novotný, Lenka Horváthová, Ivan Hrdý, Eva Kutějová, Robert P. Hirt, T. Martin Embley, Jiří Janata, Jan Tachezy

**Affiliations:** 1 Department of Parasitology, Faculty of Science, Charles University in Prague, Prague, Czech Republic; 2 Institute of Microbiology, Academy of Sciences of the Czech Republic, Prague, Czech Republic; 3 Institute for Cell and Molecular Biosciences, Newcastle University, Newcastle upon Tyne, United Kingdom; 4 Institute of Molecular Biology, Slovak Academy of Sciences, Bratislava, Slovak Republic; Washington University School of Medicine, United States of America

## Abstract

Mitochondrial processing peptidases are heterodimeric enzymes (α/βMPP) that play an essential role in mitochondrial biogenesis by recognizing and cleaving the targeting presequences of nuclear-encoded mitochondrial proteins. The two subunits are paralogues that probably evolved by duplication of a gene for a monomeric metallopeptidase from the endosymbiotic ancestor of mitochondria. Here, we characterize the MPP-like proteins from two important human parasites that contain highly reduced versions of mitochondria, the mitosomes of *Giardia intestinalis* and the hydrogenosomes of *Trichomonas vaginalis*. Our biochemical characterization of recombinant proteins showed that, contrary to a recent report, the *Trichomonas* processing peptidase functions efficiently as an α/β heterodimer. By contrast, and so far uniquely among eukaryotes, the *Giardia* processing peptidase functions as a monomer comprising a single βMPP-like catalytic subunit. The structure and surface charge distribution of the *Giardia* processing peptidase predicted from a 3-D protein model appear to have co-evolved with the properties of *Giardia* mitosomal targeting sequences, which, unlike classic mitochondrial targeting signals, are typically short and impoverished in positively charged residues. The majority of hydrogenosomal presequences resemble those of mitosomes, but longer, positively charged mitochondrial-type presequences were also identified, consistent with the retention of the *Trichomonas* αMPP-like subunit. Our computational and experimental/functional analyses reveal that the divergent processing peptidases of *Giardia* mitosomes and *Trichomonas* hydrogenosomes evolved from the same ancestral heterodimeric α/βMPP metallopeptidase as did the classic mitochondrial enzyme. The unique monomeric structure of the *Giardia* enzyme, and the co-evolving properties of the *Giardia* enzyme and substrate, provide a compelling example of the power of reductive evolution to shape parasite biology.

## Author Summary

In classic model organisms, cleavage of signals that are required to deliver nuclear-encoded proteins to mitochondria is mediated by an enzyme comprising two different subunits, called α or β, neither of which is functional by itself. Here, we have characterized a novel enzyme that functions in the mitosome, a highly reduced mitochondrion, of the pathogenic protist *Giardia intestinalis*. The *Giardia* enzyme is unique among eukaryotes because it has undergone reductive evolution to function efficiently as a single β-subunit monomer. We also show that the recent claim that the equivalent enzyme in the hydrogenosome, another type of reduced mitochondrion of the human parasite *Trichomonas vaginalis*, functions as a homodimer of two β-subunits, is not supported. The *Trichomonas* enzyme requires both an α- and a β-subunit to function most efficiently. Computational analysis of the *Giardia* and *Trichomonas* enzymes reveals that their structures and surface charge distributions have co-evolved to match the peculiar properties of the targeting signals that they process. The *Giardia* mitosome is an ideal model for studying the limits of mitochondrial reductive evolution and, because it makes cofactors that are essential for *Giardia* survival, is a potential therapeutic target for this important human parasite.

## Introduction

The acquisition of the mitochondrial endosymbiont and its evolution into the mitochondrion were key events in the evolution of eukaryotes [1]. During this process, most of the protomitochondrial genome was either lost or transferred to the nucleus of the host cell [2]. As a consequence, most mitochondrial proteins are host-nuclear encoded and must be specifically targeted to the organelle where they function. In the best understood system, N-terminal extensions attached to mitochondrial matrix proteins are specifically recognised by receptors on the mitochondrial surface, and the preproteins are subsequently imported by translocases of the outer and inner mitochondrial membranes [3]. A final step in the import process is the removal of the N-terminal extension, by the mitochondrial processing peptidase (MPP) [4], to prevent it from interfering with protein function and/or stability [5]. The MPP comprises a catalytic βMPP subunit that binds a zinc cation using amino acid residues of the conserved motif H*XX*EH*X*_76_E [6], and a regulatory αMPP subunit with a flexible glycine-rich loop that is important for substrate recognition [7]. The two subunits together form a negatively charged cavity that accommodates and immobilizes presequences during processing [6]. The activity of MPP thus requires the cooperative action of both subunits; neither subunit is functional alone [6],[8].

Mitochondrial targeting presequences are characterized by the ability to form a positively charged amphipathic alpha helix, but otherwise show little primary sequence conservation [6]. Their most prominent common feature is the presence of a cleavage motif, which determines the peptide bond to be cleaved by the processing peptidase. The cleavage motif includes a positively charged residue, typically arginine, at the -2 or -3 position from the cleavage site (P_2_ or P_3_), which is followed by hydrophobic (P_1_′) and hydrophilic (P_2_′, P_3_′) residues [9]. Mutational analyses indicate that the P_2_ (P_3_) arginine plays a key role in the recognition of the processing site by MPP and interacts with the glutamate of the βMPP active site [9]. In addition, there are one or more basic amino acid residue(s) N-terminally distal from the processing site that bind to acidic residues of the MPP cavity and stabilize the substrate-MPP complex [10].

Mitosomes and hydrogenosomes are highly reduced versions of mitochondria that are found in diverse parasitic or free-living unicellular eukaryotes inhabiting oxygen-poor or intracellular niches [1]. The organelles found in human parasites *Giardia intestinalis* and *Trichomonas vaginalis* lack a genome so all of their proteins are encoded by the nuclear genome and must be imported [1]. Some hydrogenosomal and mitosomal proteins have N-terminal extensions that are reminiscent of the presequences that direct proteins into mitochondria and they contain distinguishable cleavage motifs [11],[12]. This suggests that the *Giardia* and *Trichomonas* organelles may also contain an MPP-like enzyme. A single gene coding for a putative processing peptidase has been found in the genome of *G. intestinalis* [13] and the gene product has been shown to localize in mitosomes [14]. The primary structure of GPP is highly divergent from mitochondrial homologues, with only 13.1% identity and 29.7% similarity to the βMPP of *Saccharomyces cerevisiae*. A single gene for a βMPP homologue (20.9% identity and 42.9% similarity to *S. cerevisiae* βMPP) was also recently identified in the genome of *T. vaginalis* [15]. In this case, functional data were presented suggesting that the hydrogenosomal processing peptidase (βHPP) functioned as a homodimeric enzyme [15]. No αMPP homologue was detected, although a protein rich in glycine amino acid residues (GRLP), that shares a limited similarity with the glycine-rich loop of αMPP, was located to *T. vaginalis* hydrogenosomes. However, GRLP was reported not to stimulate βHPP activity *in vitro* [15].

The progenitor of MPP was probably a monomeric α-proteobacterial peptidase, similar to the recently described *Rickettsia prowazekii* processing peptidase (RPP) [16]. During the evolution of mitochondria, gene duplication and subunit specialization gave rise to the heterodimeric α/βMPP, which is now present in the mitochondrial matrix or integrated as the core I and II subunits of the cytochrome *bc1* complex in the inner mitochondrial membrane [6]. The single subunit structure of GPP and HPP [15] could thus reflect retention of the ancestral form of organization, or reductive evolution from the classic MPP heterodimer. It has also been suggested that the *Giardia* protein may have had a separate origin by lateral gene transfer from a bacterium other than the mitochondrial endosymbiont [13]. Here we show that GPP functions as a monomer consisting of a single βMPP homologue while HPP, like classical MPP, is fully active only upon heterodimerization of an α and β subunit. Based upon phylogenetic and functional analyses we infer that the unique monomeric structure of the *Giardia* mitosomal processing peptidase GPP, is the result of reductive, substrate-driven evolution from a heterodimeric progenitor enzyme.

## Results/Discussion

### Phylogenetic analyses of GPP, βHPP and GRLP

**Figure 1:**
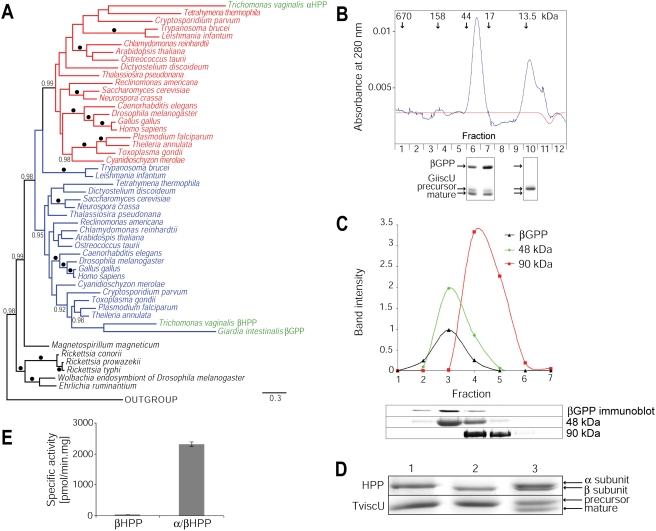
Phylogenetic and functional characterization of βGPP and α/βHPP. (A) Bayesian phylogenetic analysis of MPP-like protein sequences using a model [17] that allows for across-tree changes in protein amino acid composition. Scale bar indicates estimated substitutions per site. Posterior probabilities of 1.0 are shown as black dots on nodes, and those greater than 0.95 are shown as values. Bacterial MPP homologues are shown in black, αMPP in red and βMPP in blue. *Trichomonas* α- and βHPPs and *Giardia* βGPP are highlighted in green. Only α-proteobacterial relationships are shown for bacteria. The fit between the model and the data is shown in Fig. S1 and the full tree with additional details are shown in Fig. S2. (B) Protein size exclusion chromatography of purified recombinant βGPP showing that it elutes as a single peak between 17 and 44 kDa. The activity of βGPP was assayed for cleavage of the targeting presequence of GiiscU for each fraction and the products were separated by SDS-PAGE. Shift in protein mobility indicates cleavage of a presequence. βGPP activity was only detected in fractions from the central peak. (C) Separation of proteins from a mitosome-rich fraction on a sucrose gradient along with molecular size markers. Bands on the immunoblot and SDS-PAGE were quantified by densitometry. The calculated molecular mass of the βGPP monomer is 44.5 kDa. (D) Processing activity of the αHPP-His (lane 1), βHPP-His (lane 2) and corresponding α/βHPP heterodimer (lane 3) with TviscU, showing that the α- and β-subunits are both required for activity. (E) Specific activities were also determined for the βHPP subunit and the α/βHPP heterodimer with a fluorescent substrate based on the *T. vaginalis* adenylate kinase presequence (n = 3, mean values with s.d.) The activity of the βHPP subunit by itself is at the limit of detection for this assay.

To investigate the origins of the MPP-like proteins of *Giardia* and *Trichomonas* and the *Trichomonas* GRLP we carried out a phylogenetic analysis. As these proteins are heterogeneous for their amino acid compositions, and because a failure to accommodate such heterogeneity can lead to incorrect trees [17], we used a recently described node-discreet-compositional-heterogeneity method to analyze the data [17]. A heterogeneous model comprising 10 composition vectors was found sufficient to produce data of similar composition to the original sequences, as judged by Bayesian posterior predictive simulation [17] (Fig. S1). Phylogenetic analyses using this model support the hypothesis that GPP, βHPP and βMPP share a common origin. This result contrasts with a previous analysis, using a poorly fitting composition homogeneous model, when GPP was reported to have no phylogenetic affinity with either MPP or the α-proteobacteria [13]. The position of the GPP among βMPP, together with the presence of the catalytic motif H*XX*EH*X*_76_E, are consistent with the protein being a βMPP-like peptidase (βGPP), and not an αMPP-like protein as currently annotated [13]. Importantly, these data, together with the absence of an αMPP-like protein coding sequence on the *Giardia* genome, support the hypothesis that the single subunit structure of GPP results from reductive evolution including loss of an αMPP-like subunit. The alternative possibility, that the simple GPP structure reflects retention of the ancestral form of organization, is not supported by our analyses. Our results suggest that αMPP and βMPP probably arose once by a primordial gene duplication at the base of eukaryotes, and that all MPP-like proteins share common ancestry with single subunit enzymes from α-proteobacteria, consistent with an origin from the mitochondrial endosymbiont (Fig. 1A). Notably, our analyses show that the *T. vaginalis* GRLP is part of the αMPP clade, suggesting that, contrary to previous claims [15], *T. vaginalis* may possess a functional homologue (GRLP) of αMPP (henceforth αHPP).

### GPP functions as a β monomer while HPP forms an α/β heterodimer

**Figure 2:**
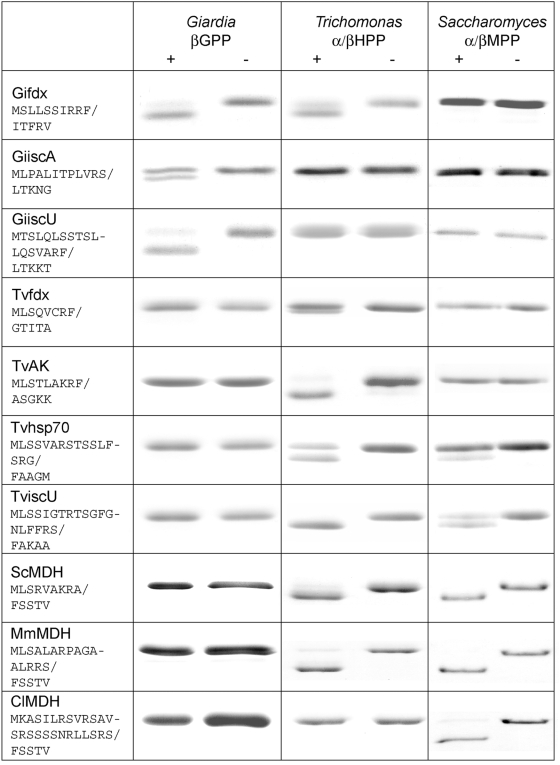
Comparative processing of mitosomal, hydrogenosomal and mitochondrial proteins by βGPP, α/βHPP and *S. cerevisiae* α/βMPP. The sequence of the demonstrated N-terminal mitochondrial, mitosomal or hydrogenosomal targeting presequences is indicated for each substrate protein with / indicating the cleavage site. Processing of *Giardia intestinalis* mitosomal presequences (Gifdx, [2Fe2S] ferredoxin; GiiscA and GiiscU, metallochaperones involved in FeS cluster assembly), *Trichomonas vaginalis* hydrogenosomal presequences (Tvfdx, [2Fe2S] ferredoxin; TvAK, adenylate kinase; Tvhsp70, heat shock protein 70; TviscU, metallochaperone involved in FeS cluster assembly) and mitochondrial presequences (ScMDH, *Saccharomyces cerevisiae* malate dehydrogenase; MmMDH, *Mus musculus* MDH; ClMDH, *Citrullus lanatus* MDH) was tested. Reaction products were separated by SDS-PAGE. Shift in protein mobility indicates cleavage of a targeting presequence. The sites of cleavage indicated by slashes in left column were determined by N-terminal amino acid sequencing. Substrates were incubated with (+) or without (−) the corresponding protease.

**Figure 3:**
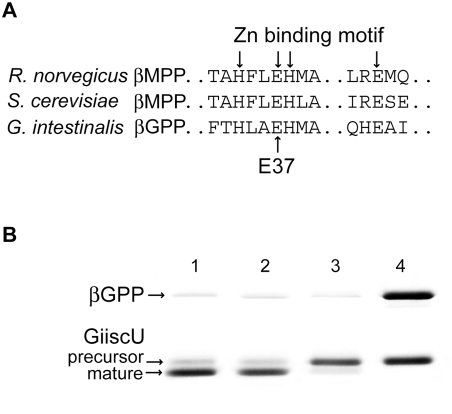
The βGPP is a metallopeptidase with a similar cleavage mechanism to α/βMPP. (A) Alignment of βGPP and βMPP subunit showing the conserved zinc-binding motif. (B) Effect of protease inhibitors and mutation of E37 on the activity of βGPP. Lane 1: βGPP+GiiscU showing cleavage to produce the mature protein; lane 2: βGPP+GiiscU+serine and cysteine protease inhibitors showing no inhibition; lane 3: βGPP+GiiscU+EDTA showing inhibition of cleavage; lane 4: Mutant βGPP in which E37 was mutated to glutamine+GiiscU, showing that the mutation of a key residue for βMPP activity also eliminates βGPP activity.

To investigate the functionality of the βGPP, βHPP and αHPP-like proteins, we expressed them in *E. coli*. The recombinant βGPP processed the N-terminal extensions of *Giardia* mitosomal ferredoxin (Gifdx) and the iron-sulphur cluster scaffold proteins (GiiscU and GiiscA). The processing activity was demonstrated as a shift in the substrate gel mobility and the cleavage sites were identified by N-terminal amino acid sequencing of the cleaved products (Fig. 2). The activity of the recombinant βGPP was inhibited by the chelator EDTA, and activity was also lost when the first glutamate of the H*XX*EH*X*_76_E motif was mutated to glutamine (Fig. 3). These data indicate that the βGPP is an active metallopeptidase with a similar cleavage mechanism to MPP [6]. Like the rickettsial homologue of MPP [16], βGPP was active as a monomer, which was demonstrated by size exclusion chromatography of recombinant βGPP as well as by analysis of βGPP from a mitosome-rich fraction separated on a sucrose gradient under native conditions (Fig. 1B, C). Importantly, kinetic parameters of monomeric βGPP (V_max_ = 0.27 µM/min; K_m_ = 8.4 µM, Fig. S3) were comparable to those published for the heterodimeric MPP of *Neurospora crassa* [8]. It has recently been suggested that the *T. vaginalis* HPP functions as a homodimer of two identical βHPP subunits [15], so we investigated the activity of βHPP with- and without αHPP. Unlike for βGPP, no activity for βHPP alone could be detected by gel shift assay (Fig. 1D), but a small amount of activity was observed when a highly sensitive fluorometric assay was used [15] (Fig. 1E). However, the processing activity measured by this assay increased by almost two orders of magnitude when the βHPP was associated with the αHPP-like protein, indicating that–like classic MPP–the *T. vaginalis* HPP functions most efficiently as a heterodimer (Fig. 1E).

### Mitosomal and some hydrogenosomal targeting presequences lack distal positively charged residues

To further investigate the structure-function relationships of the GPP, HPP and MPP, we screened *in silico* the *G. intestinalis* and *T. vaginalis* proteomes for putative mitosomal and hydrogenosomal N-terminal presequences (Tables S1 and S2), which were then analyzed for structural elements known to mediate substrate-MPP interactions. In particular, we searched for the positively charged residues proximal to the cleavage site (P_2_ or P_3_), and those which are N-terminally distal from the processing site. The distance between the proximal and distal group was defined to be at least 3 amino acid residues [18],[19]. *Giardia* mitosomal presequences were predicted in three of nine putative mitosomal proteins (Table S2). All of these presequences possess the proximal P_2_ arginine within a conserved cleavage motif [(ARV)R(F/L)(L/I)T], but the distal positively charged residues are absent (Table S2). The lengths of the *Giardia* mitosomal presequences that have been experimentally verified are 10, 12 and 18 amino acid residues. The majority of the *in silico* predicted *Trichomonas* hydrogenosomal presequences (147) resemble the *Giardia* pattern; having a length of 4 to 21 amino acid residues, possessing a P_2_ arginine within a cleavage motif, and lacking the distal positively charged residues. However, we also detected 79 putative hydrogenosomal presequences, of 10 to 24 amino acids, that–like classic mitochondrial sequences–do contain distal arginines or lysines at position P_6_–P_22_.

### Properties of MPP, GPP and HPP reflect the character of their respective substrates

To compare the specificities of the βGPP, α/βHPP and yeast α/βMPP in vitro, we tested their activity on a selection of mitosomal, hydrogenosomal and mitochondrial substrates (Fig. 2). The βGPP cleaved only its own mitosomal substrates. By contrast, the α/βHPP cleaved the hydrogenosomal presequences, and the presequences of mitosomal ferredoxin and two mitochondrial substrates. The yeast α/βMPP processed all of the mitochondrial substrates and the two mitochondrial-like hydrogenosomal substrates that possess distal positively charged residues. We also tested whether we could make chimeric peptidases using a combination of hydrogenosomal and mitochondrial subunits. Interestingly, while the yeast αMPP did not interact with the *Trichomonas* βHPP, *Trichomonas* αHPP was able to form a heterodimer with yeast βMPP. However, this heterodimer did not cleave mitochondrial or hydrogenosomal substrates under our experimental conditions (data not shown).

**Figure 4:**
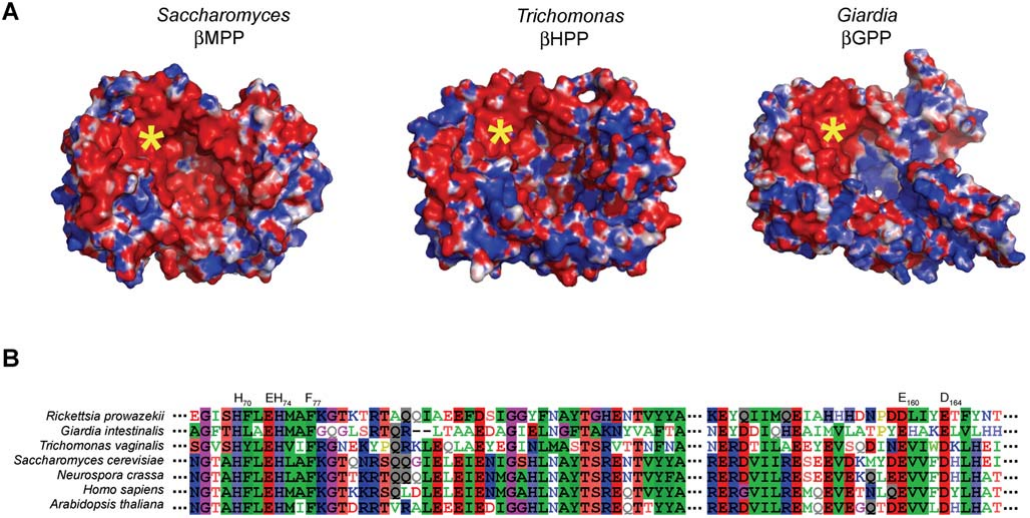
Comparative distribution of charge polarity between mitochondrial, hydrogenosomal and mitosomal peptidases. (A) Predicted charge polarity distribution of the βHPP subunit and βGPP based on the known structure and charge distribution of the *Saccharomyces cerevisiae* βMPP subunit [10]. Red and blue colours denote negative and positive charge (±5 kT/e where kT is thermal energy and e is unit charge), respectively, whereas white denote relatively non-polar regions. The yellow asterisk marks the Zn-binding region in the active site of the enzyme (shown in b). The negative charges are distributed evenly in the cavity of βMPP while in the cavity of βGPP the negative charges are concentrated mainly around the active site. The MODELLER program [29] version 9.2 was used to build 3-D models of αHPP, βHPP and βGPP. The electrostatic properties of the model were evaluated using APBS version 0.5.1 [33]. (B) Alignment of key segments where negatively charged residues of βMPP are located and known to interact with the substrate. Numbered residues are those of yeast βMPP. E_160_ and D_164_ make a salt bridge with substrate residue R-2 (P_2_) and F_77_ interacts with P_1_′ which is also often a F residue. H_70_-X-X-E_73_-H_74_ is the conserved motif of the active site.

To gain further insights into the structure-function basis of their different substrate spectra, we modelled each of the different proteins (Fig. 4 and Fig. S4), using the yeast MPP structure as a guide [10]. For yeast MPP, the substrate is first recognized by the glycine-rich loop of αMPP [6],[7] and then moved to the active site of βMPP which interacts with the substrate cleavage motif including the proximal arginine. The distal positive residues of the presequence help to stabilize the substrate-MPP complex by binding to negatively charged residues within the large polar cavity formed by the α/βMPP subunits [10]. The part of the substrate-binding cavity formed by βMPP thus displays an evenly distributed negative charge to accommodate both proximal and distal positively charged residues of mitochondrial presequences. The αMPP interacts only with the distal positive residues of longer (>20 amino acid residues) mitochondrial presequences [9],[10].

As the βGPP functions as a monomer we predict that its substrates, including the proximal arginine, interact directly with the negatively charged region of its catalytic site (Fig. 4). The rest of the predicted βGPP cavity is, unlike βMPP, positively charged, although its predicted overall fold structure still resembles that of βMPP (Fig. S4). The difference in βGPP charge distribution is compatible with the absence of distal positively charged residues in the mitosomal presequences, and, along with the absence of an αMPP-like subunit, may explain the inability of βGPP to process mitochondrial-type presequences. The simplicity of GPP is consistent with the highly reduced function of mitosomes and likely reflects (i) the paucity of proteins that are targeted to this organelle when compared with mitochondria and (ii) lack of N-terminal cleavable presequences in the majority of mitosomal proteins, including βGPP itself. As shown above (Table S2), only nine mitosomal proteins have been identified so far and these are involved either in organelle biogenesis (Gipam18, GiHsp70, GiCpn60, GPP) or the formation of Fe-S clusters (GiiscS, GiiscU, GiiscA, Gigrx, Gifdx), which is currently the only known mitosomal function for *G. intestinalis*. Of these, seven are targeted to mitosomes in the absence of a detectable N-terminal targeting signal and thus function independently of GPP. Other than these, no other homologues of mitochondrial proteins have so far been identified in the genome of *G. intestinalis* [13].

The *T. vaginalis* HPP represents an intermediate stage between GPP and MPP in terms of charge distribution and enzymatic activity. Thus, it can process presequences with- or without distal positive residues, but can only cleave the shorter mitochondrial presequences (Fig. 2). The presence of mitochondrial type presequences on hydrogenosomal proteins is consistent with the retention of the αHPP, which is likely involved in their recognition via its glycine-rich loop and/or their docking at the cleavage site.

Our phylogenetic and functional analyses show that the *Giardia* GPP is a striking example of reductive evolution from a heterodimeric to a monomeric enzyme, with properties resembling the putative ancestral α-proteobacterial enzyme, rather than the highly specialized MPP heterodimer found in well characterized mitochondria. While the principal selective pressure for the evolution of the processing peptidases is probably their ability to efficiently process substrates, the differences in the properties of the substrate presequences may also reflect the mode of their translocation across the organelle membranes [20]. In mitochondria from species across the phylogenetic tree [21], the positive residues of N-terminal presequences are recognized by the outer membrane TOM system and then the inner membrane translocase complex TIM23 [3]. Interestingly, no receptors (Tom20, Tom 22, Tom70) or components of the translocation channel of the TOM complex (Tom40, Tom5, Tom6, Tom7) have so far been identified for *G. intestinalis* [13] or *T. vaginalis* [22]. Putative core components of the TIM23 translocase (Tim23, Tim17) as well as Pam18 involved in protein transfer to the matrix have been found in *T. vaginalis*, but only Pam18 was found in *G. intestinalis* [14]. It thus appears that reductive evolution of the organelles has dramatically affected both the processing peptidases and the protein import pathway [21], with important implications for general models of mitochondrial biosynthesis, structure and function.

## Materials and Methods

### Phylogenetic analysis

Complete sequences of βGPP, βHPP, and βMPP were aligned with Muscle [23] to calculate sequence identity and similarity values. MPP, GPP and HPP sequences were aligned with Muscle [23] and analysed with Gblocks [24] to remove ambiguously aligned sites. Bayesian phylogenetic analyses were conducted using P_4_ (http://www.nhm.ac.uk/research-curation/projects/P4/index.html). The optimal substitution model for Bayesian analyses was identified by ProtTest [25] (WAG+Gamma), a polytomy prior [26], and one or more base composition vectors, which were free to vary during the chain under the NDCH model [17]. MCMC chains were run for 1,000,000 generations, sampling trees and parameters every 200 generations. Model parameter proposal tuning values were determined using the P_4_ “autoTune” method. The burn-in was identified using the method of Beiko and co-workers [27]. The base composition component of the model was tested by simulation of the base composition χ2 statistic [17] at each sampling point, resulting in a posterior predictive distribution [28] against which the statistic of the original data could be tested using tail-area probability. Composition vectors were successively added until adequate fitting of the observed data to the model was identified (see Fig. S1).

### Preparation of recombinant proteases and substrate proteins

The βGPP (NCBI accession: XP_001707100), αHPP (XP_001276882) and βHPP (XP_001316822) subunits and their substrates were expressed with hexahistidine tags in *E. coli.* An α/βHPP heterodimer was assembled from βHPP-His and non-tagged αHPP subunits by incubation of lysates of *E. coli* expressing the respective proteins for 30 min on ice in 20 mM Tris, 20 mM NaCl (pH 8.6), 1 mM MnCl_2_. All recombinant proteins were purified by nickel column chromatography (HiTrap Chelating) under native (βGPP-His, αHPP-His, βHPP-His, and α/βHPP-His) or denaturing (substrate proteins) conditions. An α/βMPP heterodimer was prepared as published [18].

### *In vitro* protease activity assays

The GPP reactions were carried out in 20 mM Tris (pH 8.0), 100 mM NaCl, 1mM MnCl_2_, 30 min at 37°C, the HPP reactions in 20 mM Tris-HCl (pH 8.6), 20 mM NaCl, 2 mM MnCl_2_, 30 min at 37°C and activity of MPP was determined in 50 mM HEPES (pH 7.4), 20 mM NaCl, 1 mM MnCl_2_, 30 min at 30°C. To identify the cleavage sites, all substrates processed by the three proteases were subjected to N-terminal protein sequencing by Edman degradation. The kinetics of GPP was determined using the method published by Arretz and co-workers [8]. For determination of the activity of the HPP subunits, purified αHPP-His and βHPP-His were incubated on ice for 30 min either alone, or mixed together with 1 mM MnCl_2_. After addition of TviscU, the reaction was allowed to proceed at 37°C for 60 min. The specific activity of HPP with a fluorescent substrate based on the presequence of TvAK [Abz-MLST LAKRF AY(NO_2_)GKKDRM] (Bachem, Switzerland) was measured at 420 nm, with an excitation wavelength of 315 nm (Infinite M200, Tecan).

### Size exclusion chromatography of purified GPP

A pre-calibrated Superdex 200 column was used to determine the molecular mass of *E. coli* produced GPP, under native conditions. Affinity purified βGPP-His in buffer of 50 mM CHES (pH 9.5), 150 mM NaCl was loaded on the column and washed (0.5 ml/min), collecting 1 ml fractions. Protein-containing fractions were assayed for βGPP activity.

### Sucrose gradient centrifugation of a *Giardia* mitosome-enriched fraction

The molecular mass of GPP expressed in *G. intestinalis* with a hemagglutinin (HA) tag was estimated under native conditions by sucrose gradient centrifugation [15]. The mitosome-enriched fraction was isolated from a *G. intestinalis* homogenate using a published method [14]. The proteins in the mitosomal-enriched fraction were then separated on a calibrated sucrose gradient [15]. Fractions were analysed by immunoblot using anti-HA antibodies. Bands visualized by alkaline phosphatase were quantified by densitometry (GS-800 Calibrated Densitometer, BioRad).

### Hydrogenosomal and mitosomal presequence identification

An application based on the NetBeans Platform (http://platform.netbeans.org) was developed to search for proteins containing N-terminal hydrogenosomal and mitosomal presequences in the predicted *T. vaginalis* (http://www.trichdb.org/trichdb/) and *G. intestinalis* (http://www.giardiadb.org/giardiadb/) proteomes, respectively. Hydrogenosmal presequences were predicted based on two main parameters extracted from 21 known hydrogenosomal presequences: (i) the cleavage site motif, specified as RXF/(ILFSAGQ) or R(FNESG)/(ILFSAGQ) (the slash indicates the cleavage site and brackets mean one residue position), and the presequence start motif defined as ML(STACGR) or MTL or MSL. In addition, tryptophan was forbidden from the presequence, the maximum presequence length was optimized to 25 residues. Any presequences with overall negative charges were excluded (the approximate presequence charge at pH7 was counted according to the Henderson-Hasselbalch equation using the following pKa values: N-terminus 8.0, lysine 10.0, arginine 12.0, histidine 6.5, glutamic acid 4.4, aspartic acid 4.4, tyrosine 10.0, and cysteine 8.5). The *G. intestinalis* proteome was searched for N-terminal presequences based on three experimentally verified mitosomal presequences of known mitosomal proteins [13] (Table S2). The parameters defined for the search were as follows: the cleavage site motif was defined as R(FS)/(IL)T, the presequence start motif as M(SLT), the maximum presequence length was set up to 20 residues, tryptophan was forbidden from the presequence. A search using parameters for prediction of hydrogenosomal presequences did not reveal additional mitosomal protein candidates.

### Protein structure prediction

The MODELLER program [29] version 9.2 was used to build 3-D models of αHPP, βHPP and βGPP. Alignments of the βGPP and βHPP with the βMPP (pdbid 1HR6) [10] and of the αHPP with the αMPP (pdbid 1HR6) [10] were carried out using the PROBCONS web service [30] and manually edited. The quality of the final model was checked using the ProCheck [31] and WhatCheck [32] programs. The electrostatic properties of the model were evaluated using APBS version 0.5.1 [33].

## Supporting Information

Figure S1 Bayesian model composition fit to the data assessed by posterior predictive simulation. Bars show the posterior distribution of χ2 for the homogeneous composition model (red) and the heterogeneous composition (NDCH) model with 10 composition vectors (green) in comparison to the statistic from the observed data. The simulated data for the NDCH model include the χ2 statistic from the observed data whereas the simulated data from the homogeneous model do not, the NDCH model thus provides a much better fit to the data. The original χ2 statistic for the data was 1292. In the simulations from the homogeneous analysis, this statistic ranged between 617 and 933 (mean = 763), while in the heterogeneous analysis (10 composition vectors) the statistic ranged between 877 and 1487 (mean = 1132 ).
(0.08 MB PDF)

Figure S2 Bayesian phylogenetic analysis of MPP-like protein sequences using the NDCH model [17] that allows for across-tree changes in protein amino acid composition. The tree is a majority rule consensus of 3,500 trees sampled from the posterior probability distribution of an MCMC with 10 across-tree composition vectors. Scale bar indicates estimated substitutions per site. Values on branches are posterior probabilities. Bacterial MPP homologues are shown in black, αMPP in red and βMPP in blue. *Trichomonas* α- and βHPPs and *Giardia* βGPP are highlighted in green.
(0.51 MB PDF)

Figure S3 The enzyme kinetics of the monomeric βGPP. The Lineweaver-Burk double reciprocal plot of reaction velocity, calculated as concentration of processed GiiscU in µM per minute versus concentration of GiiscU precursor. The least square fit line through the data intercepts x and y axes at −1/K_m_ and 1/V_max_, respectively. The kinetic parameters calculated for βGPP were: V_max_ = 1.7 µM/_min_; K_m_ = 8.4 µM; *k*cat = 17 min^−1^.
(0.10 MB PDF)

Figure S4 Tertiary structures of MPP, HPP and GPP. Homology models of βHPP and βGPP were built using the known structure of *Saccharomyces cerevisiae* βMPP, αHPP was modelled using *S. cerevisiae* αMPP. β-sheets are shown in yellow, α-helices in red, loops in grey. The glycine-rich loop of the α subunits and the zinc-biding motif of β subunits are highlighted in green. The MODELLER program [29] version 9.2 was used to build 3-D models of αHPP, βHPP and βGPP. The PROCHECK program version 3.5.4 was used to verify the validity of the model and gave a overall G-factor value of −0.12, which is well above −0.5; values below −0.5 indicates unusual structures [34]. Secondary structure prediction with PSIPRED [35] was also consistent with the modelled structure, recovering all five beta-sheets and the majority of alpha-helices (12 of 18).
(5.37 MB PDF)

Table S1 N-terminal presequences of hydrogenosomal proteins predicted in the *T. vaginalis* proteome.
(0.08 MB PDF)

Table S2 N-terminal presequences of mitosomal proteins found in *G. intestinalis* proteome.
(0.07 MB PDF)
