# Supplementary figures and images for: Reductive Evolution of the Mitochondrial Processing Peptidases of the Unicellular Parasites Trichomonas vaginalis and Giardia intestinalis
Source: PLoS Pathog. 2008 Dec 19;4(12):e1000243. doi: 10.1371/journal.ppat.1000243 (PMC2597178; doi:10.1371/journal.ppat.1000243)

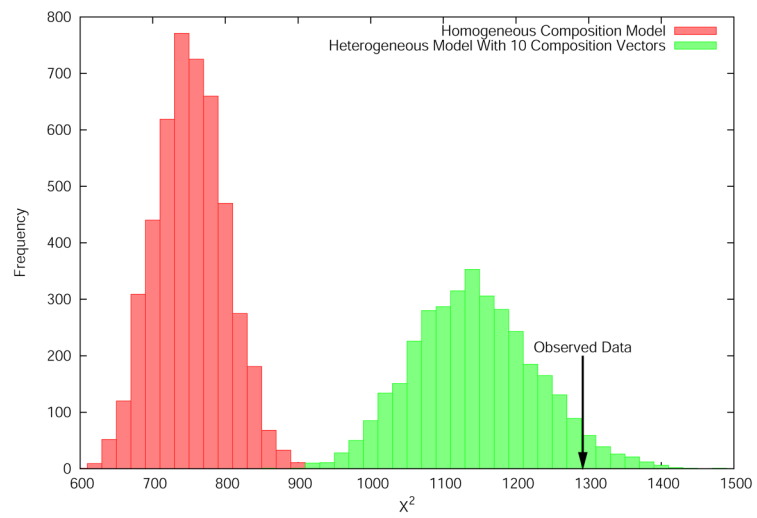

Supplement: Figure S1 — Bayesian model composition fit to the data assessed by posterior predictive simulation. Bars show the posterior distribution of χ2 for the homogeneous composition model (red) and the heterogeneous composition (NDCH) model with 10 composition vectors (green) in comparison to the statistic from the observed data. The simulated data for the NDCH model include the χ2 statistic from the observed data whereas the simulated data from the homogeneous model do not, the NDCH model thus provides a much better fit to the data. The original χ2 statistic for the data was 1292. In the simulations from the homogeneous analysis, this statistic ranged between 617 and 933 (mean = 763), while in the heterogeneous analysis (10 composition vectors) the statistic ranged between 877 and 1487 (mean = 1132 ). (0.08 MB PDF) [file ppat.1000243.s001.pdf]

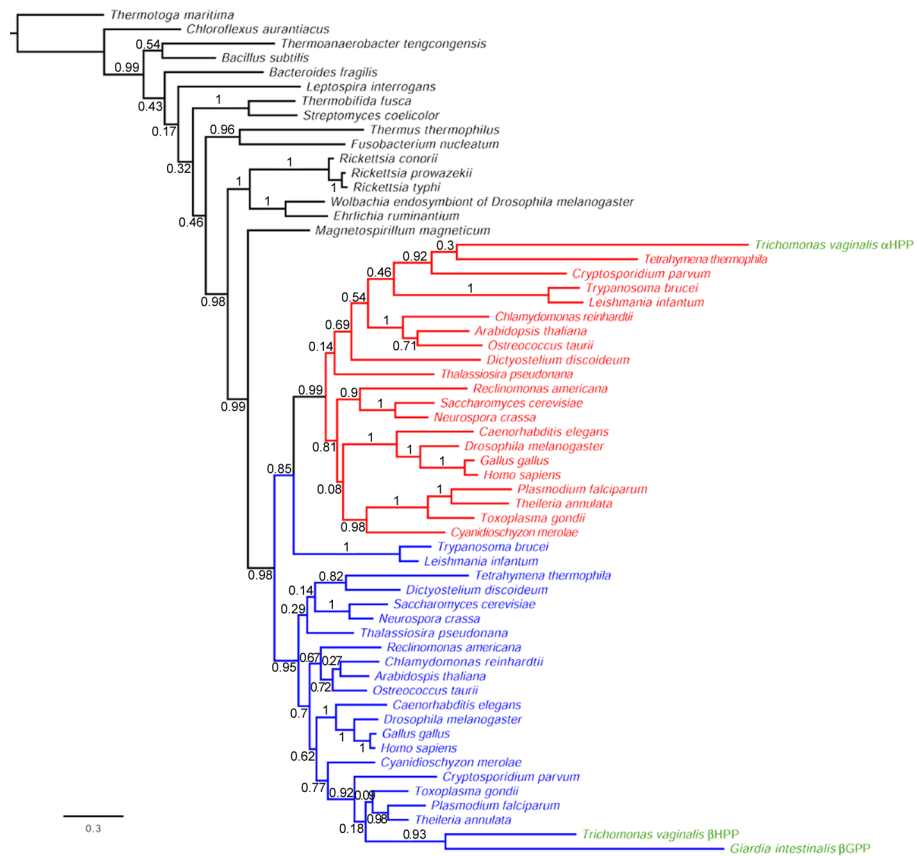

Supplement: Figure S2 — Bayesian phylogenetic analysis of MPP-like protein sequences using the NDCH model [17] that allows for across-tree changes in protein amino acid composition. The tree is a majority rule consensus of 3,500 trees sampled from the posterior probability distribution of an MCMC with 10 across-tree composition vectors. Scale bar indicates estimated substitutions per site. Values on branches are posterior probabilities. Bacterial MPP homologues are shown in black, αMPP in red and βMPP in blue. Trichomonas α- and βHPPs and Giardia βGPP are highlighted in green. (0.51 MB PDF) [file ppat.1000243.s002.pdf]

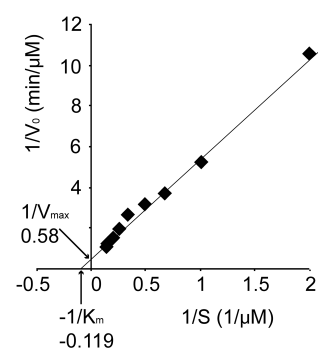

Supplement: Figure S3 — The enzyme kinetics of the monomeric βGPP. The Lineweaver-Burk double reciprocal plot of reaction velocity, calculated as concentration of processed GiiscU in µM per minute versus concentration of GiiscU precursor. The least square fit line through the data intercepts x and y axes at −1/Km and 1/Vmax, respectively. The kinetic parameters calculated for βGPP were: Vmax = 1.7 µM/min; Km = 8.4 µM; kcat = 17 min−1. (0.10 MB PDF) [file ppat.1000243.s003.pdf]
