# Supplementary material for: Reductive Evolution of the Mitochondrial Processing Peptidases of the Unicellular Parasites Trichomonas vaginalis and Giardia intestinalis
Source: PLoS Pathog. 2008 Dec 19;4(12):e1000243. doi: 10.1371/journal.ppat.1000243 (PMC2597178; doi:10.1371/journal.ppat.1000243)
Supplement: Figure S4 — Tertiary structures of MPP, HPP and GPP. Homology models of βHPP and βGPP were built using the known structure of Saccharomyces cerevisiae βMPP, αHPP was modelled using S. cerevisiae αMPP. β-sheets are shown in yellow, α-helices in red, loops in grey. The glycine-rich loop of the α subunits and the zinc-biding motif of β subunits are highlighted in green. The MODELLER program [29] version 9.2 was used to build 3-D models of αHPP, βHPP and βGPP. The PROCHECK program version 3.5.4 was used to verify the validity of the model and gave a overall G-factor value of −0.12, which is well above −0.5; values below −0.5 indicates unusual structures [34]. Secondary structure prediction with PSIPRED [35] was also consistent with the modelled structure, recovering all five beta-sheets and the majority of alpha-helices (12 of 18). (5.37 MB PDF) [file ppat.1000243.s004.pdf]

	α	β	α/β
Saccharomyces MPP	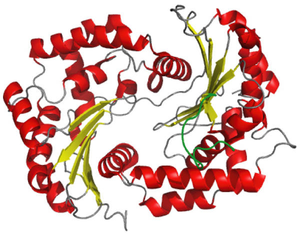	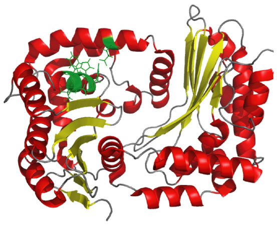	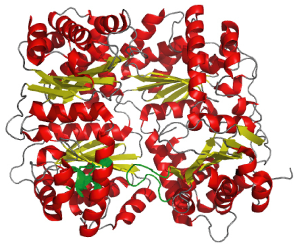
Trichomonas HPP	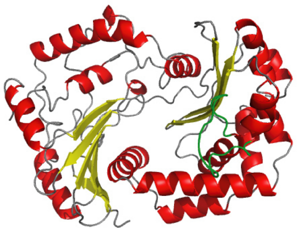	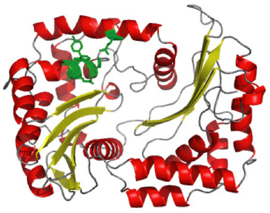	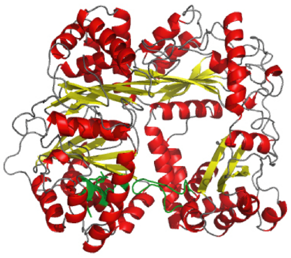
Giardia GPP	not present	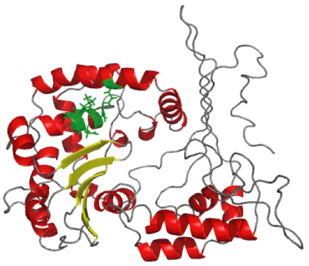	not present
