# Supplementary material for: Reductive Evolution of the Mitochondrial Processing Peptidases of the Unicellular Parasites Trichomonas vaginalis and Giardia intestinalis
Source: PLoS Pathog. 2008 Dec 19;4(12):e1000243. doi: 10.1371/journal.ppat.1000243 (PMC2597178; doi:10.1371/journal.ppat.1000243)
Supplement: Table S1 — N-terminal presequences of hydrogenosomal proteins predicted in the T. vaginalis proteome. (0.08 MB PDF) [file ppat.1000243.s005.pdf]

Table S1

N-terminal presequences of hydrogenosomal proteins predicted in the *T. vaginalis* proteome. *T. vaginalis* proteins with (A) presequences containing proximal arginine only, (B) presequences with both proximal and distal positive residues. Slash indicates predicted cleavage sites. Experimentally verified hydrogenosomal presequences are highlighted in bold.

A

Protein ID NCBI	N-terminal presequence	Presequence length	Presequence charge at pH 7.0	Annotation
TVAG_045480	MLRF/GD	4	1.909	high molecular weight subunit PW212 precursor-related protein
TVAG_336890	MLRF/GV	4	1.909	conserved hypothetical protein
TVAG_333710	MLRF/SD	4	1.909	conserved hypothetical protein
TVAG_368750	MLRG/IK	4	1.909	conserved hypothetical protein
TVAG_165200	MLRG/IK	4	1.909	neurofilament protein, putative
TVAG_165200	MLRG/IK	4	1.909	neurofilament protein, putative
TVAG_090770	MLRG/IV	4	1.909	conserved hypothetical protein
TVAG_475700	MLRN/AF	4	1.909	conserved hypothetical protein
TVAG_337780	MLRN/AL	4	1.909	conserved hypothetical protein
TVAG_495580	MLRN/FT	4	1.909	conserved hypothetical protein
TVAG_015120	MLRN/LD	4	1.909	conserved hypothetical protein
TVAG_214000	MLRN/QD	4	1.909	hypothetical protein
TVAG_499270	MLRN/QE	4	1.909	hypothetical protein
TVAG_035490	MLRS/AL	4	1.909	conserved hypothetical protein
TVAG_131910	MLRS/FT	4	1.909	conserved hypothetical protein
TVAG_465120	MLRS/IE	4	1.909	conserved hypothetical protein
TVAG_136750	MLRS/IF	4	1.909	clathrin coat adaptor ap3 medium chain, putative
TVAG_184410	MLRS/LA	4	1.909	hypothetical protein
TVAG_304640	MLRS/LG	4	1.909	beige/BEACH domain containing protein
TVAG_106590	MLRS/SI	4	1.909	conserved hypothetical protein
TVAG_254890	MLRNF/GK	5	1.909	pyruvate:ferredoxin oxidoreductase E
TVAG_242960	MLRNF/GK	5	1.909	pyruvate:ferredoxin oxidoreductase B2 [1]

**TVAG_230580**	**MLRNF/SK**	**5**	**1.909**	**pyruvate:ferredoxin oxidoreductase B1** [2]
TVAG_210030	MLRSF/AK	5	1.909	OsmC-like protein
**TVAG_198110**	**MLRSF/GK**	**5**	**1.909**	**pyruvate:ferredoxin oxidoreductase A** [2]
TVAG_441970	MLGRF/IT	5	1.909	von Willebrand factor type A domain containing protein
TVAG_593050	MLGRS/SD	5	1.909	hypothetical protein
TVAG_266350	MLRRN/IK	5	2.909	conserved hypothetical protein
TVAG_045030	MLSRE/GI	5	0.912	conserved hypothetical protein
TVAG_223970	MLSRG/LG	5	1.909	conserved hypothetical protein
TVAG_178120	MLSRS/II	5	1.909	protein phosphatase-7, putative
TVAG_146620	MLSRS/IL	5	1.909	protein phsophatase-2A, putative
TVAG_459960	MLSRS/IL	5	1.909	protein phosphatase-1, putative
TVAG_316160	MLSRS/IL	5	1.909	protein phosphatase-5, putative
TVAG_153840	MLSRS/LS	5	1.909	conserved hypothetical protein
TVAG_252470	MTLRF/QN	5	1.909	conserved hypothetical protein
TVAG_252480	MTLRF/QN	5	1.909	conserved hypothetical protein
TVAG_252460	MTLRF/QS	5	1.909	conserved hypothetical protein
TVAG_339330	MLSRIF/GK	6	1.909	conserved hypothetical protein
TVAG_457260	MLSRLF/ST	6	1.909	conserved hypothetical protein
TVAG_099790	MLSRVF/SL	6	1.909	conserved hypothetical protein
TVAG_069270	MLTRQF/ST	6	1.909	CMGC family protein kinase
TVAG_437150	MLTPRS/LR	6	1.909	conserved hypothetical protein
TVAG_205530	MSLKRG/AG	6	2.908	conserved hypothetical protein
TVAG_043080	MSLVRS/GR	6	1.909	conserved hypothetical protein
TVAG_163820	MSLYRF/SV	6	1.908	hypothetical protein
TVAG_485180	MTLPRS/SI	6	1.909	conserved hypothetical protein
TVAG_424100	MLSSIRF/AI	7	1.909	conserved hypothetical protein
TVAG_266800	MSLARRN/QL	7	2.909	conserved hypothetical protein
TVAG_377880	MTLGKRE/IR	7	1.911	hypothetical protein
TVAG_267050	MSLMHRVF/GK	8	2.149	conserved hypothetical protein
TVAG_432650	MLAAVSRS/SA	8	1.909	NifU-like protein, putative
TVAG_191660	MLALTSRN/FA	8	1.909	groes chaperonin, putative
TVAG_154730	MLASASRF/AT	8	1.909	Iron-sulfur flavoprotein
TVAG_078730	MLASISRS/AV	8	1.909	Ferredoxin 7
TVAG_099490	MLASLSRN/FG	8	1.909	ROK family protein

TVAG_041340	MLATFARN/FA	8	1.909	chaperonin, 10 kDa family protein
TVAG_183850	MLCSIQRS/IT	8	1.878	amidinotransferase family protein
TVAG_182150	MLSGFSRS/IM	8	1.909	Frataxin[3]
TVAG_114560	MLSGFSRS/LM	8	1.909	Frataxin[3]
TVAG_158970	MLSGIYRS/FS	8	1.908	protein phosphatase 2C, putative
TVAG_421060	MLSISSRS/GS	8	1.909	C-MYB, putative
TVAG_253010	MLSKVERS/AA	8	1.911	conserved hypothetical protein
**TVAG_003900**	**MLSQVCRF/GT**	**8**	**1.878**	**Ferredoxin 1 (Tvfdx)**[4]
TVAG_076230	MLSQVGRF/FA	8	1.909	mrp, putative
TVAG_393850	MLSSAARS/IA	8	1.909	Acetyl-CoA hydrolase, putative
TVAG_164890	MLSSASRS/IA	8	1.909	Acetyl-CoA hydrolase, putative
TVAG_381290	MLSSIARS/LS	8	1.909	Hsp20/alpha crystallin family protein
TVAG_456770	MLSSIIRS/FA	8	1.909	HesB-like domain containing protein
TVAG_412560	MLSSISRS/IT	8	1.909	OsmC-like protein
TVAG_444140	MLSSTSRF/AT	8	1.909	Iron-sulfur flavoprotein
TVAG_299570	MLSYVHRF/IC	8	2.148	conserved hypothetical protein
TVAG_373690	MLTQAFRS/FS	8	1.909	HesB-like domain containing protein
TVAG_071030	MSLKAKRN/IY	8	3.907	hypothetical protein
TVAG_361540	MLSQAFRAF/AQ	9	1.909	HesB-like domain containing protein
TVAG_205390	MLTSIGRYF/AK	9	1.908	small GTP-binding protein, putative
**TVAG_133030**	**MLAAYGHRF/QT**	**9**	**2.148**	**hydrogenosomal NADH dehydrogenase 51 kDa subunit**
**TVAG_318670**	**MLAGDFSRN/LH**	**9**	**0.912**	**succinate thiokinase alpha-chain** [5]
TVAG_392320	MLASFGLRF/AT	9	1.909	groes chaperonin, putative
TVAG_449080	MLSILLARF/SL	9	1.909	conserved hypothetical protein
TVAG_152030	MLSILLARF/SL	9	1.909	conserved hypothetical protein
TVAG_040870	MLSLALFRF/GS	9	1.909	Clan CA, family C1, papain-like cysteine peptidase
TVAG_213140	MLSLCQTRF/AS	9	1.878	Ferredoxin 3
TVAG_393380	MLSLFLTRF/SS	9	1.909	conserved hypothetical protein
TVAG_211590	MLSSCAKRG/LK	9	2.877	protein ssnA, putative
TVAG_066380	MLSSQFVRF/AD	9	1.909	metalo-beta-lactamase superfamily protein
**TVAG_259190**	**MLSSSFARN/FN**	**9**	**1.909**	**succinate thiokinase beta-chain** [6]
**TVAG_047890**	**MLSSSFERN/LH**	**9**	**0.912**	**succinate thiokinase alpha-chain** [5]
**TVAG_165340**	**MLSSSFERN/LH**	**9**	**0.912**	**succinate thiokinase alpha-chain** [5]

TVAG_146730	MLSSVGSRF/AA	9	1.909	Iron-sulfur flavoprotein
TVAG_040030	MLSSVGSRF/AA	9	1.909	Iron-sulfur flavoprotein
**TVAG_489800**	**MLSTLAKRF/AS**	**9**	**2.908**	**TvAK** [7]
TVAG_036010	MLSTSSARSFS/AL	9	1.909	hydrogenosomal oxygen reductase
TVAG_217870	MLTSAFKRF/AG	9	2.908	mrp, putative
TVAG_370510	MLTSIPSRF/AA	9	1.909	multimeric flavodoxin domain containing protein, putative
TVAG_096520	MTLNQTTRF/AS	9	1.909	pyruvate:ferredoxin oxidoreductase D
TVAG_205570	MLSHLTNRPF/IN	10	2.149	conserved hypothetical protein
TVAG_215080	MLSSFLSRTF/AN	10	1.909	conserved hypothetical protein
TVAG_242760	MLSSFLSRTF/AN	10	1.909	conserved hypothetical protein
TVAG_589340	MSLSKSEREF/II	10	0.913	conserved hypothetical protein
TVAG_068150	MLCSFSNSRF/FK	10	1.878	Ferredoxin 6
TVAG_292710	MLCSVSNYRF/FK	10	1.877	Ferredoxin 4
TVAG_354390	MLGTSKSYRN/LY	10	2.907	conserved hypothetical protein
**TVAG_144730**	**MLSNGSFARN/FN**	**10**	1.909	**succinate thiokinase beta-chain** [6]
TVAG_399860	MLSQCSPLRF/GS	10	1.878	Ferredoxin 2
TVAG_329120	MLTQNIPQRF/GK	10	1.909	CAMK family protein kinase
TVAG_125860	MSLIATPSRS/FA	10	1.909	conserved hypothetical protein
TVAG_132440	MSLQSKNVRS/SS	10	2.908	Alanine aminotransferase, putative
TVAG_113640	MTLPARQLRN/GL	10	2.909	alkohol dehydrogenase, putative
TVAG_302980	MTLPQRQIRN/GL	10	2.909	alkohol dehydrogenase, putative
TVAG_236210	MTLPQRQIRN/GL	10	2.909	conserved hypothetical protein
**TVAG_296220**	**MLASVNTSRFF/AR**	**11**	**1.909**	**hydrogenosomal NADH dehydrogenase 24 kDa subunit**
TVAG_183500	MLSASSNFARN/FN	11	1.909	succinate thiokinase beta-chain [1]
TVAG_062660	MLSFFFSLSRS/AC	11	1.909	hypothetical protein
TVAG_112950	MLSLFISLSRS/AD	11	1.909	conserved hypothetical protein
TVAG_385350	MLSSISSFARF/AL	11	1.909	thioredoxin family protein
TVAG_086470	MLSSISSFSRF/AL	11	1.909	thioredoxin family protein
**TVAG_412220**	**MLTSVSLPVRN/IC**	**11**	**1.909**	**malic enzyme D** [8]
**TVAG_416100**	**MLTSVSYPVRN/IC**	**11**	**1.908**	**malic enzyme C** [8]
TVAG_491540	MLSIELQIGRQF/QD	12	0.912	conserved hypothetical protein
TVAG_183790	MLASSVAAPVRN/IC	12	1.909	malic enzyme [1]
TVAG_094800	MLSILFTECLRS/SR	12	0.881	conserved hypothetical protein

TVAG_009360	MLSLLLITLSRS/AV	12	1.909	dnaK protein
TVAG_445730	MLTSLNTFGLRF/SF	12	1.909	chaperonin, 10 kDa family protein
**TVAG_238830**	**MLTSSVNFPARE/LS**	**12**	**0.912**	**malic enzyme B** [8]
**TVAG_340290**	**MLTSSVSLPARE/LS**	**12**	0.912	**malic enzyme H** [9]
**TVAG_267870**	**MLTSSVSVPVRN/IC**	**12**	**1.909**	**malic enzyme A** [8]
TVAG_485280	MLGIFFSIASCRS/LR	13	1.878	3D domain containing protein
TVAG_150360	MLSHISHSSFLRF/FS	13	2.39	thiogalactoside transacetylase, putative
TVAG_453700	MSLTTSDAQKLRE/LF	13	0.913	EF hand family protein
TVAG_335420	MLSSSSPLIVVLRS/LN	14	1.909	TolA protein, putative
TVAG_269160	MSLSCSVGLSEKRN/SN	14	1.88	hypothetical protein
TVAG_458080	MSLSPQQFFEIIRG/GK	14	0.912	conserved hypothetical protein
TVAG_037570	MLASTGINSTANILRN/IT	16	1.909	64kDa iron hydrogenase, putative
TVAG_010180	MLSFLSYFALSAVTRN/GK	16	1.908	DnaJ domain containing protein
TVAG_019190	MLTISHSGLPSSFLRF/LT	16	2.149	DnaJ domain containing protein
TVAG_000350	MTLPFLPFLLYNKYRF/LE	16	2.906	conserved hypothetical protein
TVAG_361590	MLATASASTSNILRN/IT	17	1.909	64kDa iron hydrogenase, putative
TVAG_393400	MLSLLFAQLAVSIRG/QK	17	1.909	conserved hypothetical protein
TVAG_269930	MLSVFIHTTNRSFRN/LI	17	3.149	conserved hypothetical protein
TVAG_107350	MLCQLTVIQSLLQNRVF/IN	17	1.878	conserved hypothetical protein
TVAG_382990	MLANPGTNGLLPMIVRF/LN	17	1.909	conserved hypothetical protein
TVAG_216970	MLSCLFLIGVLQSLERN/GL	17	0.881	conserved hypothetical protein
TVAG_182340	MLSSLDCLPSTFMRTF/AE	18	0.881	co-chaperone GrpE family protein
TVAG_553580	MLGACIMTGMPYTKGARF/LS	18	2.876	conserved hypothetical protein
TVAG_486320	MLSIILSHIACETDPQQIIRN/ID	21	0.124	conserved hypothetical protein
TVAG_424790	MLSILLNHITCETDPQQILRN/ID	21	0.124	conserved hypothetical protein

B

Protein ID NCBI	N-terminal presequence	Presequence length	Presequence charge at pH 7.0	Annotation
TVAG_379550	MSLKTISRLF/AY	10	2.908	tyrosine aminotransferase, putative
TVAG_480640	MLSVRKGERF/LI	10	2.911	conserved hypothetical protein
TVAG_361080	MTLRTLCERF/AA	10	1.881	hypothetical protein
TVAG_026600	MLRSVCISSRG/GF	11	2.878	conserved hypothetical protein
TVAG_321030	MLSALKSGIRF/SS	11	2.908	CoA binding domain containing protein
TVAG_060450	MLRHLTVRPGRF/SD	12	4.149	conserved hypothetical protein
TVAG_088050	MLSKASSAFVRS/FV	12	2.908	chaperonin, putative
TVAG_277380	MLTTFGKHFARG/FA	12	3.148	mrp, putative
TVAG_542680	MLAAEFKKRYGRE/LI	13	2.911	conserved hypothetical protein
TVAG_213670	MLALFLQRANLRE/SS	13	1.912	conserved hypothetical protein
TVAG_070600	MLGKVMMSKVERS/AA	13	2.91	conserved hypothetical protein
TVAG_203620	MLSKQASSAFIRS/FV	13	2.908	chaperonin 60 putative
TVAG_475950	MSLRIHKLSNYRF/GA	13	4.147	hydrolase, NUDIX family protein
TVAG_492000	MLRKLCFKAPVPRS/QS	14	4.876	conserved hypothetical protein
TVAG_090870	MLRSLQLSFRQLRG/IN	14	3.909	beige/BEACH domain containing protein
**TVAG_167250**	**MSLIEAAKHFTRAF/AK**	**14**	**2.151**	**Hsp60**[10]
TVAG_204990	MSLFCEPFRYEICRF/AN	15	0.852	conserved hypothetical protein
TVAG_399620	MLALAFLASSRTLHRS/IV	16	3.149	conserved hypothetical protein
TVAG_433130	MLATCGRHLNSSFARSF/AK	16	3.119	heat shock protein, putative
TVAG_277950	MLGLRTPPEHKSSVRS/GV	16	3.151	conserved hypothetical protein
**TVAG_237140**	**MLSSVARSTSSLFSRG/FA**	**16**	**2.909**	**Tvhsp70**
TVAG_340390	MLSSVGKTSGSLFFRS/FQ	16	2.908	heat shock protein 70 (HSP70)-4, putative
TVAG_030930	MSLKHINLTRYDERRS/SN	16	3.152	conserved hypothetical protein
TVAG_135950	MLSTKTSPFFPGYDFRS/AT	17	1.91	ef-hand domain (C-terminal) containing protein, putative
TVAG_308130	MLTLVPHRRIPMSKERS/QS	17	4.151	WD repeat-containing protein slp1, putative
TVAG_143400	MLSQPQPRKKTVRQCGRF/SI	18	5.876	conserved hypothetical protein
TVAG_356810	MLSSICRFGHSMRRHERE/IK	18	3.364	nitroimidazole resistance protein, putative
TVAG_005890	MLSSISRLGHVMRRGDRE/IK	18	3.154	nitroimidazole resistance protein, putative
TVAG_478310	MLTSKHDIHPNKYNELRS/IY	18	2.392	conserved hypothetical protein
TVAG_494010	MSLFSFNSMKHYLLKFRYF/IL	19	4.145	HEAT repeat family protein

TVAG_559350	MSLSKSERESIILNDARG/IQ	18	0.916	conserved hypothetical protein
TVAG_466520	MSLSKSERESIILNNARG/IQ	18	1.913	conserved hypothetical protein
TVAG_471430	MSLSKSERKDVVLNDARG/IQ	18	1.915	conserved hypothetical protein
TVAG_352770	MSLSKSERKSIILNDARG/IQ	18	2.912	conserved hypothetical protein
TVAG_104990	MSLSKSERQSIILHDARG/IQ	18	2.153	conserved hypothetical protein
TVAG_504860	MSLSKSKRESIILNDARG/IQ	18	2.912	conserved hypothetical protein
TVAG_250290	MSLSKSKRESIILNDARG/IQ	18	2.912	conserved hypothetical protein
TVAG_106920	MTLSKSERESIILNDARG/IQ	18	0.916	conserved hypothetical protein
TVAG_146780	MLAAVKNSTFGFGNFFIRS/FA	19	2.908	conserved hypothetical protein
TVAG_322470	MLALIAFARNPNYYNLFRF/IK	19	2.907	conserved hypothetical protein
TVAG_270230	MLRTLCQVRQNLPRYIQRS/FD	19	4.877	conserved hypothetical protein
TVAG_352670	MLRTLYQVRQNLPRNIQRS/FD	19	4.908	conserved hypothetical protein
TVAG_161150	MLRTLYQVRQNLPRSIQRS/FD	19	4.908	conserved hypothetical protein
TVAG_148060	MLRTLYQVRQNLPRSIQRS/FD	19	4.908	conserved hypothetical protein
TVAG_103700	MLRTLYQVRQNLPRYIQRS/FD	19	4.907	conserved hypothetical protein
TVAG_418580	MLRTLYQVRQNLPRYMQRS/FD	19	4.907	conserved hypothetical protein
**TVAG_008840**	**MLSSIGTRTSGFGNLFFRS/FA**	**19**	**2.909**	**TviscU**
TVAG_074640	MLTSLCMSNLGPHREGFRG/LP	19	2.121	glucosylceramidase, putative
TVAG_421230	MSLFKLIFALFISVISERG/IF	19	1.911	conserved hypothetical protein
TVAG_163470	MSLQEANGRLLEARAKQRE/LK	19	1.916	conserved hypothetical protein
TVAG_385950	MSLSSYIIHSFKHLLDPRF/GE	19	2.39	protein phosphatase-1, putative
TVAG_053240	MTLLSHLLLKNREQDMCRN/LK	19	2.123	conserved hypothetical protein
TVAG_010980	MTLVFLSLLLTDARSFRRE/AL	19	1.914	hypothetical protein
TVAG_460050	MLAKLLSFITNASPAKEYRN/IE	20	2.909	conserved hypothetical protein
TVAG_300690	MLSFLFLFISGKNITISKRG/ST	20	3.907	conserved hypothetical protein
TVAG_007300	MLTARGSTVMIRIKRTTGRS/FI	20	5.908	ubiquitin family protein
TVAG_425510	MLTKKLYRTRSTSTSSAVRG/SQ	20	5.906	conserved hypothetical protein
TVAG_140830	MSLPRQFDRNGRIHQSGSRG/SL	20	4.152	conserved hypothetical protein
TVAG_207600	MSLRSSRSSTKSTKSQVSRS/IT	20	5.907	hypothetical protein
TVAG_291910	MSLSSQTSSRRSSTNSVTRS/ST	20	3.909	conserved hypothetical protein
TVAG_072420	MSLYDEYAIHIPKSPHRRRS/AI	20	3.392	conserved hypothetical protein
TVAG_258310	MTLYRYNSQKQIVEVNLKRG/SY	20	3.908	conserved hypothetical protein
TVAG_449880	MLRSRKSSKDPQNNRRRVTRHF/LS	22	8.15	conserved hypothetical protein
TVAG_327760	MLSSPQKFAANVLLFNASARN/SG	21	2.908	Iron-sulfur flavoprotein

TVAG_452330	MSLTPRIGQLYLSGIKKRERN/SN	21	4.909	conserved hypothetical protein
TVAG_090660	MLRRSTCDANHIRQFFLSKHRYF/AR	23	5.359	conserved hypothetical protein
TVAG_085490	MLRRSTCDANHIRQFFLSKHRYF/AR	23	5.359	conserved hypothetical protein
TVAG_000900	MLRRSTCDANHIRQFFLSKHRYF/AR	23	5.359	conserved hypothetical protein
TVAG_228260	MLSSCATRRLKTIVGNGKLIRS/LN	22	5.876	amidohydrolase family protein
TVAG_160480	MSLGVSHPKLSPAQQVNQLGRN/ID	22	3.148	conserved hypothetical protein
TVAG_160860	MSLKEENEKLRNKIQQLSLQRN/QE	22	2.914	conserved hypothetical protein
TVAG_199590	MTLSKAFSAPDSVEVASYMIRS/SI	22	0.912	conserved hypothetical protein
TVAG_447950	MTLSKAFSAPDSVEVASYMIRS/SI	22	0.912	conserved hypothetical protein
TVAG_304780	MLSNIIAISFAMKSQENIKHIRE/SD	23	2.152	conserved hypothetical protein
TVAG_197430	MLTGIDLIIHCCKNDNVKILNRE/LM	23	1.094	ankyrin repeat-cotaining protein, putative
TVAG_130280	MLGIKFGNSTIVVHHIADTRRERN/AI	24	3.394	heat shock protein 70kD, putative
TVAG_174040	MLGIKFGNSTIVVHHISDTRSERN/AI	24	2.394	heat shock protein, putative
TVAG_111940	MLRTLYQVRQNLPRYIQRSFDKRN/AE	24	5.909	conserved hypothetical protein
TVAG_049690	MLRYPFTPNKPYTALALNFTFPRF/FD	24	3.906	thiamin pyrophosphokinase, putative

Table S1 References

1. Hirt RP, Noel CJ, Sicheritz-Ponten T, Tachezy J, Fiori PL (2007) *Trichomonas vaginalis* surface proteins: a view from the genome. Trends Parasitol 23: 540-547.

2. Hrdý I, Müller M (1995) Primary structure and eubacterial relationships of the pyruvate, ferredoxin oxidoreductase of the amitochondriate eukaryote *Trichomonas vaginalis*. J Mol Evol 41: 388-396.

3. Doležal P, Dancis A, Lesuisse E, Šuťák R, Hrdý I et al. (2007) Frataxin, a conserved mitochondrial protein, in the hydrogenosome of *Trichomonas vaginalis*. Eukaryot Cell 6: 1431-1438.

4. Johnson PJ, d'Oliveira CE, Gorrell TE, Müller M (1990) Molecular analysis of the hydrogenosomal ferredoxin of the anaerobic protist *Trichomonas vaginalis*. Proc Natl Acad Sci U S A 87: 6097-6101.

5. Lahti CJ, Bradley PJ, Johnson PJ (1994) Molecular characterization of the alpha-subunit of *Trichomonas vaginalis* hydrogenosomal succinyl-Coa synthetase. Mol Biochem Parasitol 66: 309-318.

6. Lahti CJ, d'Oliveira CE, Johnson PJ (1992) Beta-succinyl-coenzyme-A synthetase from *Trichomonas vaginalis* is a soluble hydrogenosomal protein with an amino-terminal sequence that resembles mitochondrial presequences. J Bacteriol 174: 6822-6830.

7. Lange S, Rozario C, Müller M (1994) Primary structure of the hydrogenosomal adenylate kinase of *Trichomonas vaginalis* and its phylogenetic relationships. Mol Biochem Parasitol 66: 297-308.

8. Hrdý I, Müller M (1995) Primary structure of the hydrogenosomal malic enzyme of *Trichomonas vaginalis* and its relationship to homologous enzymes. J Eukaryot Microbiol 42: 593-603.

9. Dyall SD, Yan W, Delgadillo-Correa MG, Lunceford A, Loo JA et al. (2004) Non-mitochondrial complex I proteins in a hydrogenosomal oxidoreductase complex. Nature 431: 1103-1107.

10. Bui ETN, Bradley PJ, Johnson PJ (1996) A common evolutionary origin for mitochondria and hydrogenosomes. Proc Natl Acad Sci U S A 93: 9651-9656.
