# Supplementary material for: Reductive Evolution of the Mitochondrial Processing Peptidases of the Unicellular Parasites Trichomonas vaginalis and Giardia intestinalis
Source: PLoS Pathog. 2008 Dec 19;4(12):e1000243. doi: 10.1371/journal.ppat.1000243 (PMC2597178; doi:10.1371/journal.ppat.1000243)
Supplement: Table S2 — N-terminal presequences of mitosomal proteins found in G. intestinalis proteome. (0.07 MB PDF) [file ppat.1000243.s006.pdf]

Table S2

N-terminal presequences of mitosomal proteins found in *G. intestinalis* proteome.

Protein ID NCBI	N-terminal presequence	presequence length	Presequence charge at pH 7.0	Annotation
gi:157815925	MSLLSSIRRF/IT	10	2.997	Gifdx [1]
gi:159115366	MLPALITPLVRS/LT	12	1.997	GiiscA†
gi:159117498	MTSLQLSSTSLLQSVARF/LT	18	1.997	GiiscU [1]
gi:159119780		0		GiiscS [1]
gi:159113748		0		GPP [1]
gi:159119748		0		Gihsp70 [2]
ctg02_23-5-32114-31794*		0		Gipam18 [1]
gi:159110554		0		Gicpn60 [2]
gi:159117945		0		Gigrx†

* Open reading frame ID from GiardiaDB database (http://giardiadb.org/giardiadb/).
† Experimentally verified (data not shown).

Table S2 References

1. Doležal P, Šmíd O, Rada P, Zubáčová Z, Bursac D, Šuťák R, Nebesářová J, Lithgow T, Tachezy J (2005) *Giardia* mitosomes and trichomonad hydrogenosomes share a common mode of protein targeting. Proc Natl Acad Sci U S A 102: 10924-10929.

2. Regoes A, Zourmpanou D, Leon-Avila G, van der Giezen M, Tovar J, Hehl AB (2005) Protein import, replication, and inheritance of a vestigial mitochondrion. J Biol Chem 280: 30557-30563.
